# Acupuncture for post anaesthetic recovery and postoperative pain: study protocol for a randomised controlled trial

**DOI:** 10.1186/1745-6215-15-292

**Published:** 2014-07-21

**Authors:** Johannes Fleckenstein, Petra I Baeumler, Caroline Gurschler, Tobias Weissenbacher, Michael Simang, Thorsten Annecke, Thomas Geisenberger, Dominik Irnich

**Affiliations:** 1 Multidisciplinary Pain Centre, Department of Anaesthesiology, University of Munich, Pettenkoferstrasse 8 A, 80336 München, Germany; 2 Department of Anaesthesiology, University of Munich, München, Germany; 3 Department of Traditional Chinese Medicine/Acupuncture, Institute of Complementary Medicine IKOM, University of Bern, Bern, Switzerland; 4 Department of Obstetrics and Gynaecology, Ludwig-Maximillians-University Hospital, München, Germany; 5 Institute for Medical Information Sciences, Biometry and Epidemiology (IBE), University of Munich, München, Germany; 6 Department of Anaesthesiology, Hospital of the City of Tuttlingen, Tuttlingen, Germany

## Abstract

**Background:**

We report on the design and implementation of a study protocol entitled *Acupuncture randomised trial for post anaesthetic recovery and postoperative pain - a pilot study* (*ACUARP*) designed to investigate the effectiveness of acupuncture therapy performed in the perioperative period on post anaesthetic recovery and postoperative pain.

**Methods/Design:**

The study is designed as a randomised controlled pilot trial with three arms and partial double blinding. We will compare (a) press needle acupuncture, (b) no treatment and (c) press plaster acupressure in a standardised anaesthetic setting. Seventy-five patients scheduled for laparoscopic surgery to the uterus or ovaries will be allocated randomly to one of the three trial arms. The total observation period will begin one day before surgery and end on the second postoperative day. Twelve press needles and press plasters are to be administered preoperatively at seven acupuncture points. The primary outcome measure will be time from extubation to ‘ready for discharge’ from the post anaesthesia care unit (in minutes). The ‘ready for discharge’ end point will be assessed using three different scores: the Aldrete score, the Post Anaesthetic Discharge Scoring System and an In-House score. Secondary outcome measures will comprise pre-, intra- and postoperative variables (which are anxiety, pain, nausea and vomiting, concomitant medication).

**Discussion:**

The results of this study will provide information on whether acupuncture may improve patient post anaesthetic recovery. Comparing acupuncture with acupressure will provide insight into potential therapeutic differences between invasive and non-invasive acupuncture techniques.

**Trial registration:**

NCT01816386 (First received: 28 October 2012)

## Background

Perioperative care has improved over the last decades. To continue this trend it is important note that the quality and speed of post anaesthetic recovery is influenced by multiple factors such as the occurrence of pain, postoperative nausea and vomiting (PONV), paralytic ileus, fatigue and sleep disturbances [1]. Hence, a multimodal approach to prevent and minimise these factors is considered to be essential in order to enhance recovery [2]. These include a series of elements such as providing the patient with thorough preoperative information and education concerning perioperative care, the use of safe and short-acting anaesthetics and optimal dynamic pain relief with minimal use of opioids, management of PONV, enteral nutrition, early mobilization and use of minimal invasive surgery. As a result of optimised anaesthetic protocols, patients might be fully awake earlier and breathe comfortably in the operating theatre [3]. As a result of a more rapid recovery, fewer patients arrive in the post anaesthesia care unit (PACU) sedated, and the period during which they are at risk for airway obstruction and hemodynamic instability is reduced [4].

For achieving an optimised management of postoperative pain and PONV, acupuncture may be a hitherto underestimated option. Interest in the use of acupuncture in anaesthesia leads back to 1971, when James Reston, a *The New York Times* columnist, introduced the western world to the ancient Chinese therapy of acupuncture [5]. Reston underwent an emergency appendectomy in China, and described acupuncture’s success in relieving his postoperative pain. From this point on, several scientists started investigating possible effects of acupuncture. Soon, Bruce Pomeranz revealed that an analgesic effect of acupuncture is mediated by the release of endorphins and can therefore be abolished by the administration of naloxone in humans [6]. Mice deficient in opiate receptors did not experience acupuncture-mediated analgesia [7], which confirms the opioid-related mechanism of acupuncture-analgesia. Soon, first articles reported on the benefits of acupuncture anaesthesia in various operative procedures in China [8,9]. As a consequence, first attempts were made to implement acupuncture in the anaesthetic framework [10], for example, as an option for sedation of the patient during tonsillectomy [11]. A new modified method of anaesthesia in open heart surgery combining acupuncture analgesia and controlled respiration was established at the Department of Anaesthesiology in Gießen, Germany [12]. Since then, general anaesthesia has markedly improved and acupuncture anaesthesia cannot be considered a first-line procedure.

However, there is evidence that acupuncture is effective in facilitating multimodal approaches in perioperative care. An approved acupuncture effect is the reduction of PONV and antiemetic consumption by stimulation of the acupuncture point PC 6 [13]. Recent reviews support the effectiveness of acupuncture regimen in reducing postoperative pain, cumulative opioid consumption or opioid related side effects [14,15]. However, trials are of varying quality and show heterogeneity with regard to the applied acupuncture treatments (acupuncture points used, time point of application). Accordingly, a meta-analysis on the effects of acupuncture on the intraoperative analgesic consumption and quality of anaesthesia revealed inconclusive results [16].

We aim to investigate the effects of a three-day permanent needle acupuncture treatment, starting in the preoperative phase at seven different acupuncture points only - five of them bilaterally - on a comprehensive set of above mentioned variables important to anaesthetic recovery (which are, preoperative anxiety, improving anaesthetic protocols, awakening, nausea and vomiting, and postoperative pain).

## Patients and methods

### Study design

**Figure 1:**
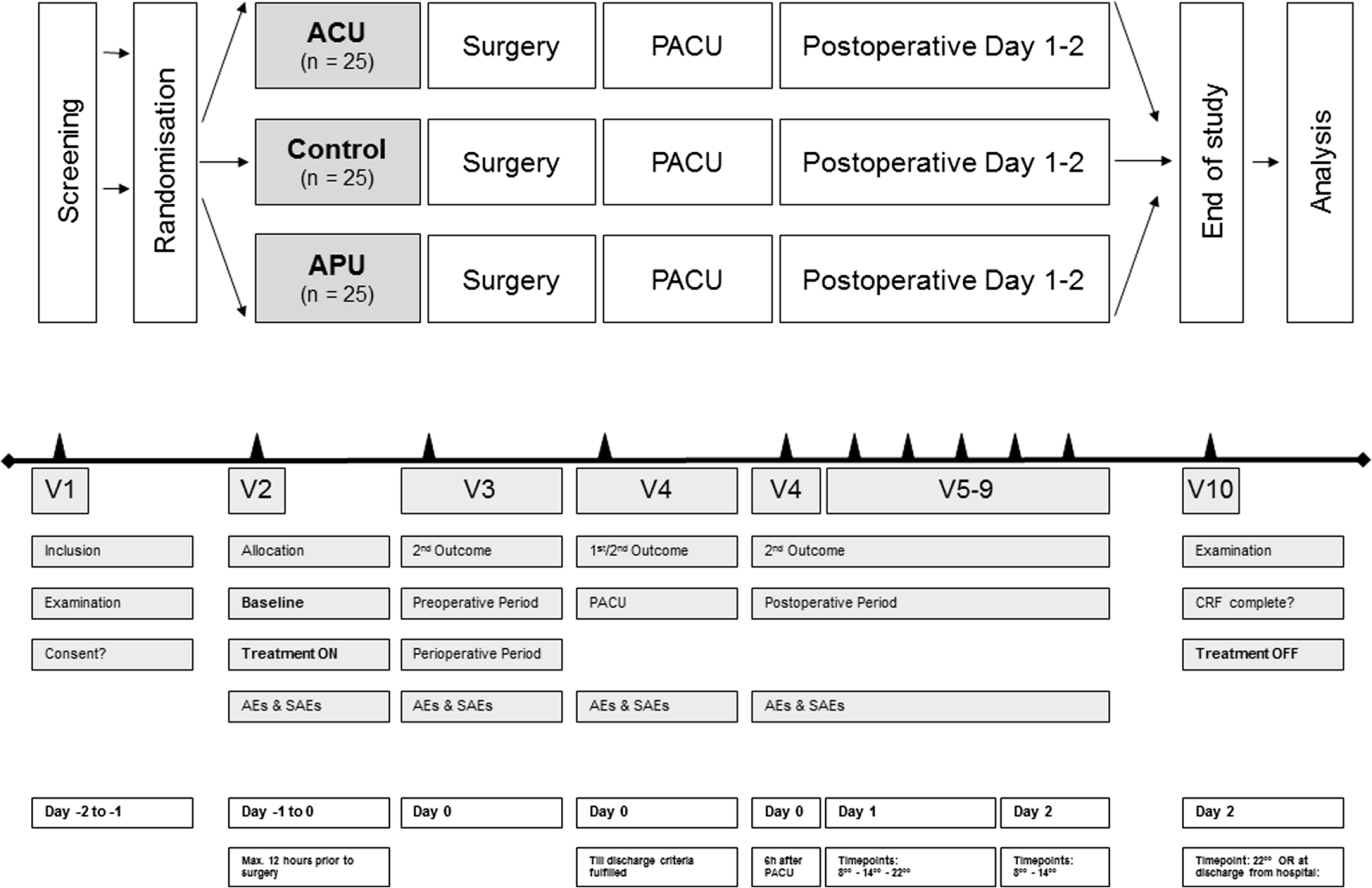
**Study design.** Patients are randomly allocated to either the group ACU (press needle acupuncture), no treatment group (control) or the group APU (press plaster acupressure). After surgery, press needles and press plasters will be kept on the patients’ skin for the next two postoperative days or until discharge from the hospital. The listed outcome measures will be assessed at the marked time points respectively.

The study is a single-center, partially double blinded, randomised pilot trial comparing (a) press needle acupuncture versus (b) no treatment versus (c) press plaster acupressure in a standard anaesthetic setting of programmed gynaecologic laparoscopic operations. Main outcome measure is the time from extubation to ‘ready for discharge’ from the PACU. Analysis of all records is performed by blinded evaluators. The total follow-up period per patient is two days (see Figure 1). The study has been approved by the Ethics Committee of the University of Munich, Germany (reference 009-12) and is in agreement with the Declaration of Helsinki (Version Fortaleza 2012). Trial registration is NCT01816386.

### Patients

For inclusion, patients must meet the following criteria:

• Age 18+

• Females scheduled for laparoscopic surgery of uterus, adnexa or ovaries

• American Society of Anaesthesiologists (ASA)-score ≤ 2

• Ability to follow study instructions and likely to attend and complete all required visits

• Written informed consent

Subjects presenting with any of the following exclusion criteria may not be included in the trial:

General exclusion criteria:

• Subject without legal capacity

• Subject who is unable to understand the nature, scope, significance and consequences of this clinical trial

• Simultaneously participation in another clinical trial or participation in any clinical trial involving administration of acupuncture within 30 days prior to inclusion

• Subjects with a physical or psychiatric condition which at the investigator’s discretion may put the subject at risk, may confound the trial results, or may interfere with the subject’s participation in this clinical trial

• Known or persistent abuse of medication, drugs or alcohol

• Current or planned pregnancy or nursing women

• Females of childbearing potential, who are not using and not willing to use medically reliable methods of contraception for the entire study duration (such as oral, injectable, or implantable contraceptives, or intrauterine contraceptive devices) unless they are surgically sterilised/hysterectomised or there are any other criteria considered sufficiently reliable by the investigator in individual cases.

Indication specific exclusion criteria:

• Surgery within the last three months

• Chronic pain > three months

• Continuous analgesic medication with opioids longer than three days

• Massive degenerative diseases

• Pre-treatment with acupuncture or trigger point injection within the last two months

### Randomised treatment allocation, blinding and sample-size estimation

Informed consent will be obtained from each participant. Subjects eligible for participation will be randomly assigned to one of the following study groups by using the Internet based randomisation software RANDOULETTE® (Institute of Medical Information Sciences, Biometry and Epidemiology, University of Munich):

• press needle acupuncture: 25 subjects

• no treatment control: 25 subjects

• press plaster acupressure: 25 subjects

Stratification for age will be performed, and an equal distribution between treatment arms (ratio of 1:1:1) will be warranted.

In this trial we will use acupressure (group C) as an intervention to compare acupuncture (group A). Patients and examiners will be blinded for the patients’ assignment to treatment arms A and C. Therefore, we use press needles (0.2 mm × 1.5 mm; Seirin New Pyonex®, Seirin Corp., Shizuoka City, Japan) in group A and non-invasive Seirin press plasters in group C - a device that has successfully been validated for blinding in acupuncture trials [17]. Press plasters do not contain a needle but a blunt knob and are in all other aspects identical to the press needles. According to group allocation, press needles or press plasters will be administered by an independent acupuncturist at the same defined acupuncture points. Instructions regarding point stimulation for subjects and study staff will also be identical. Thereby, patients and examiners will be blinded as laid down in the trial of Myazaki and colleagues who showed that neither patients nor therapists nor examiners know if a sharp tip or a blunt knob is located below the plaster. In contrast to Myazaki, patients will be allowed to stimulate their press device, so that an additional acupressure effect may be elicited, thereby enhancing the overall effects.

### Participating trial physicians

Participating trial physicians are employees of the Multidisciplinary Pain Centre, Department of Anaesthesiology, University of Munich, Germany. Their average qualification is at least equal to a third year resident in the field of anaesthesiology and specialised pain medicine. They contribute to all medical duties.

The acupuncturists are physicians at the Multidisciplinary Pain Centre, Department of Anaesthesiology, University of Munich, Germany, who have passed more than 360 hours of curricular teaching in TCM and acupuncture and who routinely use acupuncture in daily clinical practice.

### Anaesthetic proceedings

All standardised medication will be permitted according to the perioperative anaesthetic guideline and to the standard perioperative pain guideline, Department of Anaesthesiology, University of Munich, both based on general recommendations and guidelines of the German Society for Anaesthesiology (DGAI). In particular:

opioids and propofol for intraoperative anaesthesia will be administered by Target Control Infusion (TCI) according to the standard protocol. TCI is a software based on pharmacokinetic models according to Schnider, used in the Fresenius-Kabi Orchestra® Base Primea syringe pumps (Fresenius Kabi Group, Bad Homburg, Germany). It requires age, gender, height and total body weight as input for programming. The software calculates the lean body mass for the patient and determines doses and infusion rates accordingly to achieve the necessary drug concentrations at the effect site (brain). The aspired effect-site concentrations are reported in Table 1.

**Table 1:** Estimated effect-site concentrations

	**Propofol**	**Sufentanyl**
Induction of anaesthesia	4.0 to 9.0 μg/ml	0.2 to 0.4 ng/ml
Maintenance of anaesthesia	3.0 to 4.0 μg/ml	0.12 to 0.22 ng/ml

The effect-site concentrations are targeted during induction and maintenance of anaesthesia and form part of the standardised anaesthetic procedure during surgery.

To avoid postoperative pain all patients receive metamizol 2.5 g iv-infusions and to prevent PONV they receive dexamethasone 8 mg iv and for prevention of gastric stress ulcer they receive ranitidine 50 mg iv during surgery.

Postoperatively, subjects are treated for pain with metamizol (4 × 1.25 g/day) and the opioid piritramide (PCA; 2 mg each 10 minutes; maximum dosage 30 mg/4 hours). In case of nausea and vomiting or shivering, patients are treated according to the clinical standard.

Variations of this guideline based regimen are allowed if medically indicated. All concomitant therapies or medication are documented in the subjects’ clinical record file.

### Interventions

**Figure 2:**
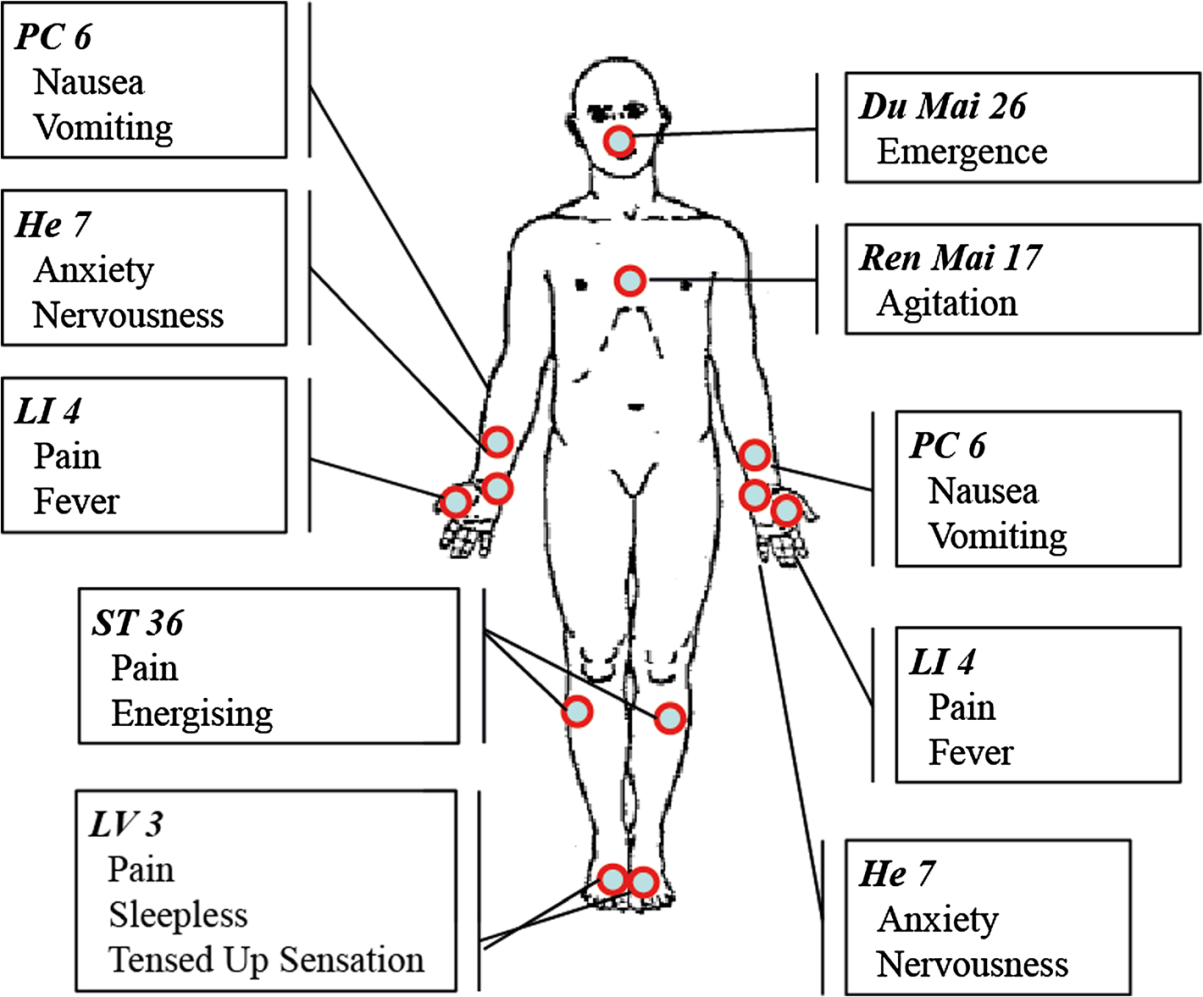
**Acupuncture points hand out.** The figure illustrates the seven acupuncture points as being distributed to the patients. The listed symptoms indicate the key indications for the respective points. The chart is handed out to the patients in the acupuncture (ACU) and acupressure (APU) groups, the given indications are thought as reminder for the patients of the possible treatment effects to be expected when self-palpating the respective points. Du 26 has to be pressed by the attending anaesthetist during emergence.

Patients in the acupuncture and the acupressure group will receive a standardised treatment with either 12 press needles (sharp tip) or 12 press plasters (blunt knob) at 7 acupuncture points (Du 26 and Ren 17 (on the middle body line), and bilateral LI 4, HE 7, LV 3, ST 36 and PC 6; see Figure 2). The needles have been provided by the SEIRIN Corporation, Shizuoka City, Japan. The point regimen is based on inquiring national experts. Application of the press needles or press plaster is performed and documented by a licensed medical acupuncturist 12 to 24 hours prior to surgery. Application time is supposed to be 72 to 96 hours. Modification will be allowed, in particular less points according to the patient’s reaction during treatment.The patient will be instructed to press the needles or plasters as often as they like, especially when they are suffering from anxiety, pain or nausea and vomiting. Patients are given an illustrated summary of the acupuncture points with the respective key symptoms, for memorization (see Figure 2). Stimulation of the press needles or press plasters has to be done at least three times a day, each time at a minimum of four different points, each for about 30 seconds. If the patient is not able to perform the daily manipulation by herself, the trial physicians are required to manipulate instead. During emergence, that is starting with the end of anaesthetic drug administration, the trial team or the anaesthetist is required to stimulate at acupuncture point Du 26.

Patients allocated to the control group will undergo the standard anaesthetic procedure only.

### Outcome measures

Main outcome measure:

Time from extubation to ‘ready for discharge’ from the PACU is defined as the latest time necessary to achieve the minimum score allowing for discharge score as assessed in three independent recovery scores. The used scores include the Aldrete score [18], the Post Anaesthetic Discharge Scoring System PADSS; [19] and the modified In-House score for outpatients of the Department of Anaesthesiology, University of Munich (for comparison see Table 2). All scores consist of five items regarding different postoperative physiologic states of the patient, which are graded from 0 to 2 (see Table 2). A summated score of 9 to 10 indicates that the patient is ready for discharge. The In-House score includes three additional ‘yes-no’ questions assessing orientation, input and output which have to be answered with ‘yes’, too. If all scores are fulfilled the patient is graded as ready for discharge.

**Table 2:** Recovery scores in the post anaesthesia care unit

	**Sub-items (0 to 2 points)**	**Aldrete score**	**PADSS**	**In-House score**
Activity	4 extremities, 2 extremities, 0 extremities	YES		
Respiration	Deep breath, Dyspnoea, Apnoea	YES		
Circulation/Vital Signs	BP ± 20 mm/20% to baseline, BP ± 20 to 50 mm/20 to 40% to baseline, BP ± 50 mm/40% to baseline	YES	YES	YES
Consciousness	Awake, Arousal on calling, Not responding	YES		
Colour	Normal, Pale, Cyanotic	YES		
Activity, Mental Status	Oriented and steady gait, Oriented or steady gait, Neither		YES	
Pain, Nausea, Vomiting	Minimal, Moderate, Severe		YES	YES, Pain and PONV are both individually assessed
Surgical Bleeding	Minimal, Moderate, Severe		YES	YES
Intake and Output	Oral fluids and voided, Oral fluids or voided, Neither		YES	
Ability to arise	By itself, no nausea, With assistance only, Not possible, Nausea			YES
Orientation^a^	Person, Place, Time			‘yes-no’ question
Intake^a^				‘yes-no’ question
Output^a^				‘yes-no’ question
*Score considered for discharge from PACU*		***≥ 9***	***≥ 9***	***≥ 9 and fulfill all ‘yes-no’ questions***

YES indicates that the item forms part of the questionnaire. Each item consists of three sub-items which are rated with 0 to 2 points with 2 points reflecting the normal physiologic state. ^a^concerns three items of the In-House score which can only be answered as ‘yes’ or ‘no’. All scores have to be 9 or greater for patients being considered ‘ready for discharge’. In case of the In-House score, the three ‘yes-no’ questions have to be answered with ‘yes’. PADSS: Post Anaesthetic Discharge Scoring System.

**Secondary outcome measure:**In the pre- the intra- and the postoperative period (in PACU and at the ward) additional outcomes will be assessed (Figure 1).

• Preoperative period

○ Preoperative anxiety (State-Trait Anxiety Inventory: STAI [20])

▪ at baseline

▪ in the morning of the surgical day

▪ immediately before induction of anaesthesia

○ Vital signs (heart rate and blood pressure) at baseline

○ Pain intensity at rest at baseline

○ Laboratory parameters according to the surgical preoperative standard at baseline

• Intraoperative period

○ Intraoperative drug consumption (analgesics, sedatives)

○ TCI effect-site concentrations

○ Vital signs (heart rate and blood pressure)

○ Bispectral Index (BIS) monitoring (recording starts prior to induction of anaesthesia)

○ Organisational time points (for example, incision to closure time; intubation to extubation time, and so on)

○ During emergence, the time point when stopping the TCI device, time to recovery of spontaneous breathing and time to extubation, time to eye opening, time to squeezing of the anaesthesiologists hand on command and orientation to time and place will be recorded if possible

○ About 15 minutes before the end of surgery, analgesia applied through the TCI pumps will be reduced (expected emergence 15 minutes) to facilitate recovery and time will be recorded. Time to extubation is defined as the time between end of anaesthesia and extubation.

• PACU

○ Time course and comparison of recovery scores (Aldrete score, PADSS, In-House scoring system)

○ Pain intensity

○ Analgesic consumption

○ Concurrent medication

○ Occurrence of surgical- or anaesthesia-related events

▪ PONV

▪ Shivering

▪ Others

○ Vital signs

• Postoperative period (on the ward)

• Patients will be visited after discharge from the PACU based on a fix scheme, which is six hours after, and three times daily on the two following postoperative days unless the patient has been previously discharged from the hospital. The following parameters will be assessed:

○ Vital signs

○ Pain intensity

○ Quantity and frequency of stimulated acupuncture points

○ Analgesic consumption

○ Concurrent medication

○ Occurrence of surgical- or anaesthesia-related events

▪ PONV

▪ Shivering

▪ Others

### Sample size calculation

No sample size calculation could be established because at the time of designing the trial there were no parameters available from prior studies. However, we estimated the sample size on the basis of an *a priori* analysis using G*Power (Version 3.1.3, University of Düsseldorf, Germany, see Table 3). We conservatively anticipated the effect size of the tested intervention to be small to medium (d = 0.4, according to Jacob Cohen [21]), which results in a sample size of 66 patients for three study groups. A further adjustment for non-parametric testing and drop outs by 15% results in a total sample size of 75 patients.

**Table 3:** Sample size calculation

F tests:	ANOVA: Fixed effects, omnibus, one-way		
Analysis:	*A priori*: Compute required sample size		
Input:	Effect size f		= 0.4
	α err probability		= 0.05
	Power (1-β err probability)		= 0.8
	Number of groups		= 3
Output:	Noncentrality parameter λ		= 10.5600000
	Critical F		= 3.1428085
	Numerator df		= 2
	Denominator df		= 63
	Total sample size		= 66
	Actual power		= 0.8

A priori analysis as calculated and exported by G*Power (Version 3.1.3, University of Düsseldorf, Germany), anticipating a small to medium effect size f = 0.4, the α-error = 0.05 and the power = 0.8.

### Data analysis

The aim of the statistical analysis is the comparison of the main outcome measure (period from extubation to ‘ready for discharge’ from PACU) between the three study groups (intervention groups acupuncture and acupressure and no treatment control). For this variable, an non-normal distribution is assumed. Hence, a nonparametric method, the Kruskal-Wallis test will be applied. *Post hoc* tests will be performed by pairwise comparisons of the three study groups by the Mann-Whitney *U*-test and subsequent Bonferroni adjustment for multiple comparison (*P*-value times three). Primary outcome is defined as the time to discharge from PACU in the press needle acupuncture group in comparison to the no treatment group.

For the secondary target variables, a method according to the distribution of data will be performed. For metric variables, the structure of a normal distribution will be tested by the Kolmogorov-Smirnov test. In case of a normal distribution, a one-way ANOVA will be calculated. Otherwise a nonparametric test for independent observations like the Kruskal-Wallis test will be carried out.

The analysis of categorical data is conducted by the chi-square goodness-of-fit test. The binary case is treated by the chi-square-test of independence.

Data analysis will be performed with the SPSS statistical software system, version 15.0 (SPSS Inc., Chicago, IL, USA) and with SAS/STAT® Software (SAS Institute Inc., Cary, NC, USA). All data entry will be carried out twice.

## Discussion

The AcuARP trial investigates the effectiveness of acupuncture on post anaesthetic recovery and its use as an adjunct therapy to improve patient’s recovery in the whole perioperative setting.

Inclusion and exclusion criteria were held pragmatic in order to facilitate screening and recruitment. The chosen trial population is homogeneous and the operations undertaken are common; hence a maximised and improved routine care would be of general interest.

Limitations include the restricted sample size as the trial was planned as a pilot study. In case of a positive study outcome, further confirmatory studies will be needed. In addition, a standardised clinical procurement, using strictly standardised anaesthesia and standardised acupuncture regimen may impede generalizability of the findings.

### Acupressure as a control procedure

The investigators have chosen acupressure as a control procedure in this trial as it provides the possibility of blinding both patients and examiners. Successful patient blinding using the press needle/plaster device has been proven by Miyazaki *et al*. [17]. Examiners will be blinded too, since they do not know if a sharp needle (acupuncture) or a blunt knob (acupressure) is located below the plaster [17].

This special trial design will allow us to assess different needle-specific treatment effects [22]: on the one hand, using a needle tip, acupuncture will be performed by penetrating the skin causing specific physiologic effects. Those may be distinguished from similar physiologic effects caused by acupressure, on the other hand, which can also be a strong stimulus by touching and kneading the skin, but without penetration. Emotional, hormonal and affiliative responses to touch have already been assured [23]. By comparing acupuncture to acupressure, this trial will provide important evidence not only about the importance of the needle stimulus of acupuncture but also about whether acupressure might also bring about a therapeutic benefit in the perioperative setting. This in particular seems to be of interest when taking into account patient populations that are at higher risk of suffering from side effects from local bleeding.

The use of press needles was also chosen for other reasons. Perioperative treatment has to be practicable, applicable, safe and scientifically approved. The practicability will be given, as press needles have only to be fixed once: for this trial after inclusion, feasible in daily routine along with the anaesthesiologic consent. The chosen acupuncture sites are not within the surgical area. Patients can easily access the needles and press them if necessary. Press plasters are water resistant and sustain up to one week. The safety of acupuncture has been described in depth, suggesting that acupuncture is free of severe side effects and being considered a safe intervention [24]. Blunt rather than sharp knobs are even less traumatic. There is a small number of trials dealing with the safety of press needles; one long-term observation over one year in cancer patients suggests a ratio of 10% of minor side effects, mostly rashes [25]. Furthermore, press needles are short (1.7 mm) and needling sites are located in a safe distance from inner organs. Thus, severe adverse events such as pneumothorax or injury of inner organs are avoided. The scientific proof was previously subject to other trials, suggesting that it is worth using acupuncture in the perioperative setting [14,15].

### Study design

We have chosen to apply acupuncture or acupressure as interventions in a three-armed double blinded randomised controlled trial.

First, acupuncture in its philosophic meaning is not primarily supposed to ‘cure’ illness - the underlying idea is that acupuncture may harmonise *Qi*, which is a Chinese concept of vitality or energy, and other tensions of the human body which can lead to feelings of pleasantness, and the alleviation of respective symptoms [26]. Several trials have shown its use different settings, for example in pain at obstetric delivery [27], chronic pain conditions [28], nausea and vomiting [13], seasonal allergic rhinitis [29], dysmenorrhoea [30], and so on. Hence acupuncture is known to alleviate primary symptoms.

Second, previous trials showed that single acupuncture points exist which are sensitive to alleviate specific symptoms relevant to the perioperative period, such as pericardium 6 (PC 6) in the treatment of postoperative nausea and vomiting [13]. Other reports suggest Governing Vessel 26 (Du 26) may be successful in shock resuscitation [31] or the area between Large intestine 8 and 10 (LI 8 and LI 10) in acute tonsillitis and pharyngitis [32]. Acupressure at Stomach 36 (ST 36) was able to shorten the time to first flatus passage, oral liquid intake, and improve gastrointestinal function in patients after abdominal surgery [33].

Third, several trials could already demonstrate its effectiveness in reducing preoperative anxiety [34], the amount of anaesthetic medications [14] or postoperative pain [15]. Most of these trials have been performed using ear acupuncture and only a minority using traditional acupuncture.

All these key facts regarding acupuncture taken together suggest that it may be a useful adjunct in the perioperative period. Thus, our aim was to develop an effective and easily applicable acupuncture regimen for the complementary support of post anaesthetic recovery. Emphasis should focus on applicability, which is to reduce the number of needling events: therefore we choose the use of press needles in this trial, providing availability of the treatment for the patients during the whole perioperative period. Regarding an effective acupuncture, we have chosen acupuncture points which are supposed to alleviate perioperative symptoms. The decision has been made on the basis of a consensus process taking historic transmission but also scientific knowledge into account.

### Anaesthetic regimen

The choice of a strictly standardised anaesthetic regimen allows for comparability of all intra- and postoperative measures. The decision to conduct the study within the gynaecologic theatre was on the one hand due to the above mentioned uniform study population, on the other hand driven by the fact that the anaesthetic regimen in this clinic is extended for the use of TCI. TCI systems for dosage of propofol have been available since 1997, were initially developed for adult patients, with the dosage based on the basis of simulated concentration of the drug in the plasma for review: [35]. In the following, taking into account the rate at which the drug enters the biophase of other tissues, that is the brain, the systems were adjusted to the concentration in the so-called effector sites. Today, the systems give the possibility to programme the dosage of various intravenous drugs according to different pharmacokinetic models, which, in brief, differ on the basis of research conducted to assess the suggested rates of infusion. The two systems mainly used are the pharmacokinetic models for propofol as established by Marsh [36] and Schnider [37]. The Marsh model operates with fixed rate constants, whereas compartment volumes and clearances are weight proportional. The Schnider model was developed during combined pharmacokinetic-pharmacodynamic modelling studies, adjusting according to total weight, lean body mass, and height. It is recommended for the use in effect-site targeting mode for review: [38].

The time course of plasma/effect-site equilibration is used to describe the rate of removal of drug from the effect-site out of the body. With effect-site targeting, the TCI system manipulates the plasma concentration to achieve the effect-site concentration as rapidly as possible. When the effect-site target concentration is increased, the TCI system briefly increases the plasma concentration to an optimal level above the target effect-site concentration before temporarily stopping the infusion to allow the plasma concentration to decrease to the level of the target effect-site concentration. If the target effect-site concentration is reduced, the system stops the infusion allowing the plasma concentrations to fall, thereby generating a concentration gradient out of the effect-site, until the estimated effect-site concentration has fallen to the new target [38].

In our believe, the use of TCI is an adequate variable to monitor and compare intraoperative drug consumption when the estimated effect site concentrations is defined *a priori*, and may therefore be helpful in reducing bias from individually administered anaesthetic drugs. To our knowledge, to date, there is no better system which would improve comparability of different study groups.

This study is a three-armed, partially double blinded, randomised controlled trial to evaluate the effectiveness of acupuncture on the post anaesthetic recovery period as assessed by the time from extubation to ‘ready for discharge’ from the PACU. It can be expected to provide valuable new information on the clinical effectiveness of acupuncture improving post anaesthetic recovery parameters, which are a) preoperative anxiety relief, b) perioperative variables of anaesthesia and narcotic guidance, c) the occurrence of postoperative pain and anaesthetic side effects such as PONV or shivering. The comparison between press needle acupuncture involving a sharp needle tip and acupressure will provide important information about a) the physiologic effect of the needle stimulus compared to pressure and its impact on relieving perioperative symptoms and b) if both treatments are useful adjuncts in the routine perioperative care.

## Trial status

The status of the study at the time of first manuscript submission is ongoing, and we had included 50 patients.

## Abbreviations

ACU: acupuncture; APU: acupressure; ASA: American Society of Anaesthesiologists; BIS: Bispectral Index; DGAI: German Society for Anaesthesiology; PACU: post anaesthesia care unit; PADSS: Post Anaesthetic Discharge Scoring System; PCA: patient controlled analgesia; PONV: postoperative nausea and vomiting; STAI: State-Trait Anxiety Inventory; TCI: Target Controlled Infusion; TCM: Traditional Chinese medicine.

## Competing interests

All authors declare that they have no competing interests and did not receive any honorarium from SEIRIN® or other partners. The investigator-initiated grant received by SEIRIN® guarantees independent conceivability of the study design, its coordination, realisation and independent report of the study results.

## Authors’ contributions

JF: conception and design, funding, data collection, manuscript writing and final approval of the manuscript. PB: conception and design, data collection, manuscript writing and final approval of the manuscript. CG: design, data collection, manuscript writing and final approval of the manuscript. TW: data acquisition, critical revision and final approval of the manuscript. MS: statistical study design, critical revision and final approval of the manuscript. TA: data acquisition, critical revision and final approval of the manuscript. TG: conception and design, critical revision and final approval of the manuscript. DI: conception and design, financial support, manuscript writing, final approval of manuscript. All authors read and approved the final manuscript.
